# Supplementary material for: HKDC1 promotes tumor immune evasion in hepatocellular carcinoma by coupling cytoskeleton to STAT1 activation and PD-L1 expression
Source: Nat Commun. 2024 Feb 13;15:1314. doi: 10.1038/s41467-024-45712-2 (PMC10864387; doi:10.1038/s41467-024-45712-2)
Supplement: Supplementary file 1 — Supplementary Information [file 41467_2024_45712_MOESM1_ESM.pdf]

HKDC1 promotes tumor immune evasion in hepatocellular carcinoma by coupling cytoskeleton to STAT1 activation and PD-L1 expression

Zhang *et al.*

Inventory of Supporting Information

1. Supplementary Figures and figure legends

Supplementary Fig. 1 | HKDC1 promotes HCC progression potentially through regulation of antitumor immunity. Related to Fig. 1.

Supplementary Fig. 2 | Aberrant HKDC1 expression in cancer cells promotes tumor immune evasion by increasing exhaustion of tumor-infiltrating $CD8^+$ T cells. Related to Fig. 1.

Supplementary Fig. 3 | HKDC1 enhances PD-L1-mediated immune evasion of HCC cells independent of its hexokinase activity. Related to Fig. 2.

Supplementary Fig. 4 | HKDC1 associates with STAT1 and facilitates its phosphorylation to enhance PD-L1 expression. Related to Fig. 3.

Supplementary Fig. 5 | HKDC1 presents cytosolic STAT1 to IFNGR1 to facilitate its phosphorylation via actin cytoskeleton protein ACTA2. Related to Fig. 4.

Supplementary Fig. 6 | HKDC1 inhibition incorporating with PD-1/PD-L1 blockade enhances T cell antitumor response in HCC model mice. Related to Fig. 5.

Supplementary Fig. 7 | HKDC1 promotes tumor immune evasion in a $CD8^+$ T cell-dependent manner by coupling cytoskeleton to STAT1/PD-L1 activation.

Supplementary Fig. 8 | Gating and sorting strategies.

Supplementary Fig. 9 | Individual analysis of t-SNE.

2. Supplementary Tables

Supplementary Table 1. Targeting Sequence of shRNAs.

Supplementary Table 2. Oligonucleotide primers used for qPCR.

Supplementary Table 3. Reagents.

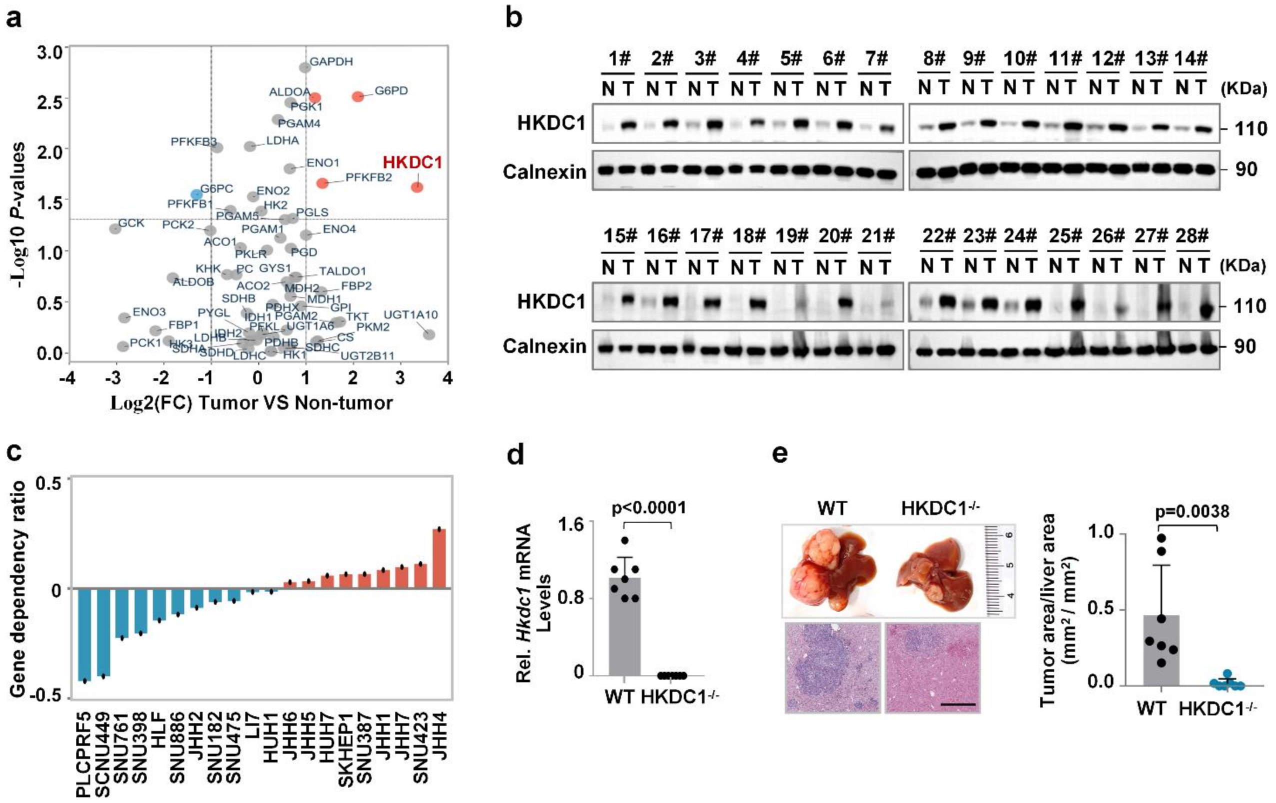

Supplementary Fig. 1 | HKDC1 promotes HCC progression potentially through regulation of antitumor immunity. Related to Fig. 1.

a, Volcano plot of the distribution of glucose-metabolic-enzyme genes. *X*-axis: glucose-metabolic-enzyme genes significantly upregulated (Log2 fold change ≥ 1) or downregulated (Log2 fold change ≤ -1) in HCC patients (Transcriptomic data from LIHC-TCGA, *n* =419 samples). *Y*-axis: -Log10 *P*-values of glucose-metabolic-enzyme genes correlation with survival of HCC patients with immunotherapy (Data from GO30140 and IMbrave150 cohorts, *n* =314 patients), and $p \leq 0.05$ means significantly associated with survival. **b**, Western blot analysis of protein levels of HKDC1 in 28 pairs of human clinically adjacent noncancerous liver tissues (N) and cancerous liver tissues (T). **c**, HKDC1 gene dependency ratio in hepatocellular carcinoma cell lines based on a large-scale CRISPR-Cas9 and RNAi screening database DepMap. **d**, qPCR analysis of mRNA levels of *Cd274* in the liver in YAP5SA-induced HCC mouse model of WT and HKDC1 KO mice (*n* = 6 male mice per group). **e**, The H.E. quantification of tumor area/liver area (mm^2/mm^2) in YAP5SA-induced HCC mouse model with indicated genotypes (*n* = 7 male mice per group) is shown on the right. Plasmids expressing YAP5SA and plasmids expressing PB transposase were injected through the tail vein into C57BL/6 mice (6-8 weeks). Images of liver and representative H.E. staining are shown on the left. Scale bars, 500 μm. Data are presented as mean ± SD (**d**,**e**). *P*-values were calculated by two-tailed unpaired Student's *t*-test (**d,e**). Source data are provided as a Source Data file.

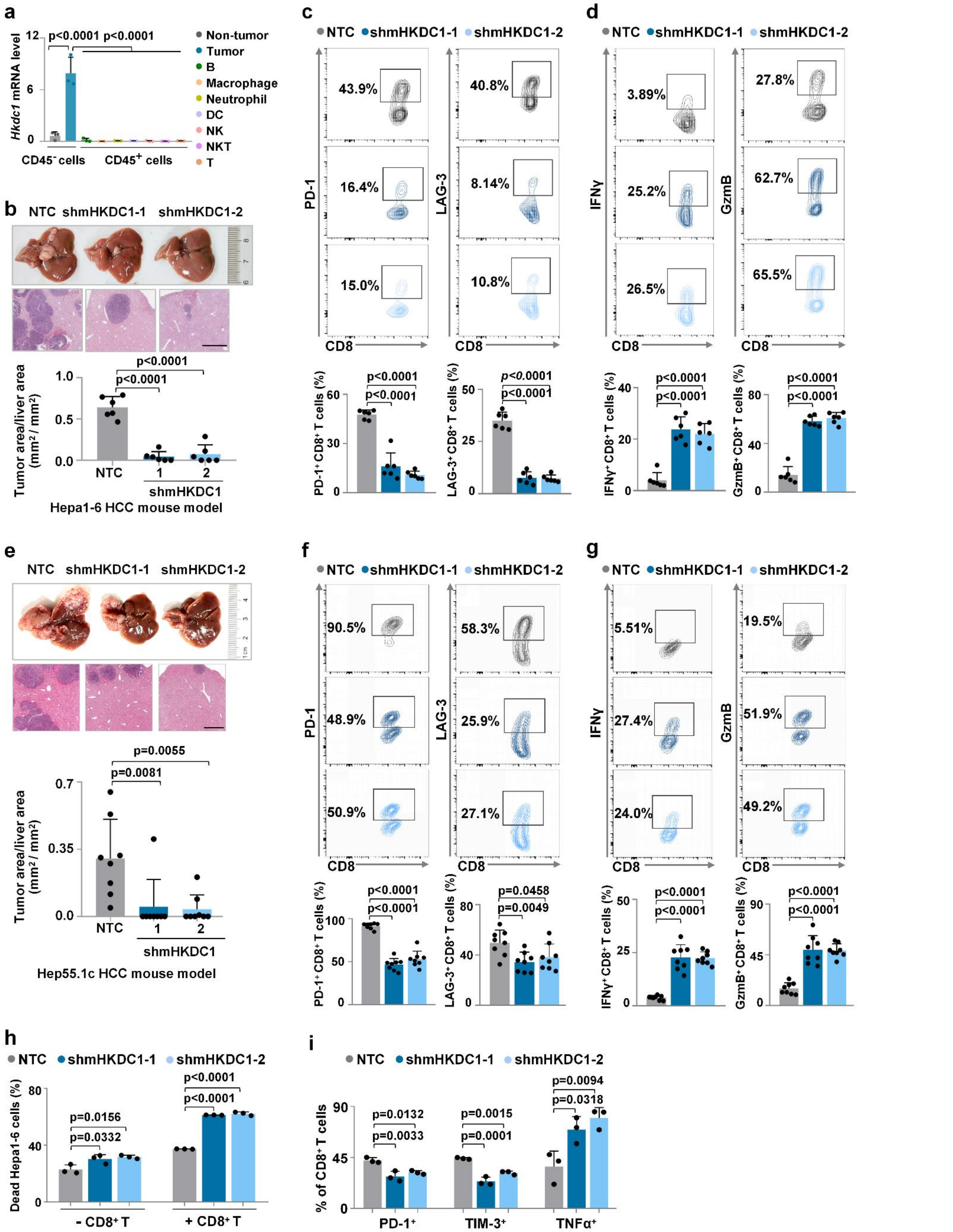

Supplementary Fig. 2 | Aberrant HKDC1 expression in cancer cells promotes tumor immune evasion by increasing exhaustion of tumor-infiltrating CD8⁺ T cells. Related to Fig. 1.

a, qPCR analysis of *Hkdc1* mRNA levels in indicated cells in YAP5SA-induced HCC mouse model

(n = 3 male mice). **b**, The H.E. quantification of tumor area/liver area (mm^2/mm^2) in Hepa1-6 xenografts with indicated genotypes (n = 6 male mice per group) is shown below. Hepa1-6 cells with indicated genotypes were injected through hepatic portal vein into C57BL/6 mice (6-8 weeks). Images of liver and representative H.E. staining are shown above. Scale bars, 500 μm. **c**, The percentage of tumor-infiltrating PD-1^+ or LAG-3^+ $CD8^+$ T cells in the hepatic portal mouse model in (**b**) was measured by flow cytometry. **d,** The percentage of tumor-infiltrating $IFN\gamma^+$ or $GzmB^+$ $CD8^+$ T cells in the hepatic portal mouse model in (**b**) was measured by flow cytometry. **e**, The H.E. quantification of tumor area/liver area (mm^2/mm^2) in Hep55.1c xenografts with indicated genotypes (n = 8 male mice per group) is shown below. Hep55.1c cells with indicated genotypes were injected through hepatic portal vein into C57BL/6 mice (6-8 weeks). Images of liver and representative H.E. staining are shown above. Scale bars, 1 mm. **f**, The percentage of tumor-infiltrating PD-1^+ or LAG-3^+ $CD8^+$ T cells in the hepatic portal mouse model in (**e**) was measured by flow cytometry. **g**, The percentage of tumor-infiltrating $IFN\gamma^+$ or $GzmB^+$ $CD8^+$ T cells in the hepatic portal mouse model in (**e**) was measured by flow cytometry. **h**, Cell death analysis of indicated Hepa1-6 cells co-cultured with activated $CD8^+$ T cells separated from mouse spleen was analyzed by flow cytometry. **i**, The percentage of PD-1^+, TIM-3^+ or $TNF\alpha^+$ $CD8^+$ T cells co-cultured with Hepa1-6 cells with indicated genotypes was analyzed by flow cytometry. Data are presented as mean ± SD (**a-g**). Data are presented as mean ± s.e.m of three biologically independent experiments (**h**, **i**). P-values were calculated by one-way ANOVA (**a-e, i, j**) or two-tailed unpaired Student's t-test (**f, g**). Source data are provided as a Source Data file.

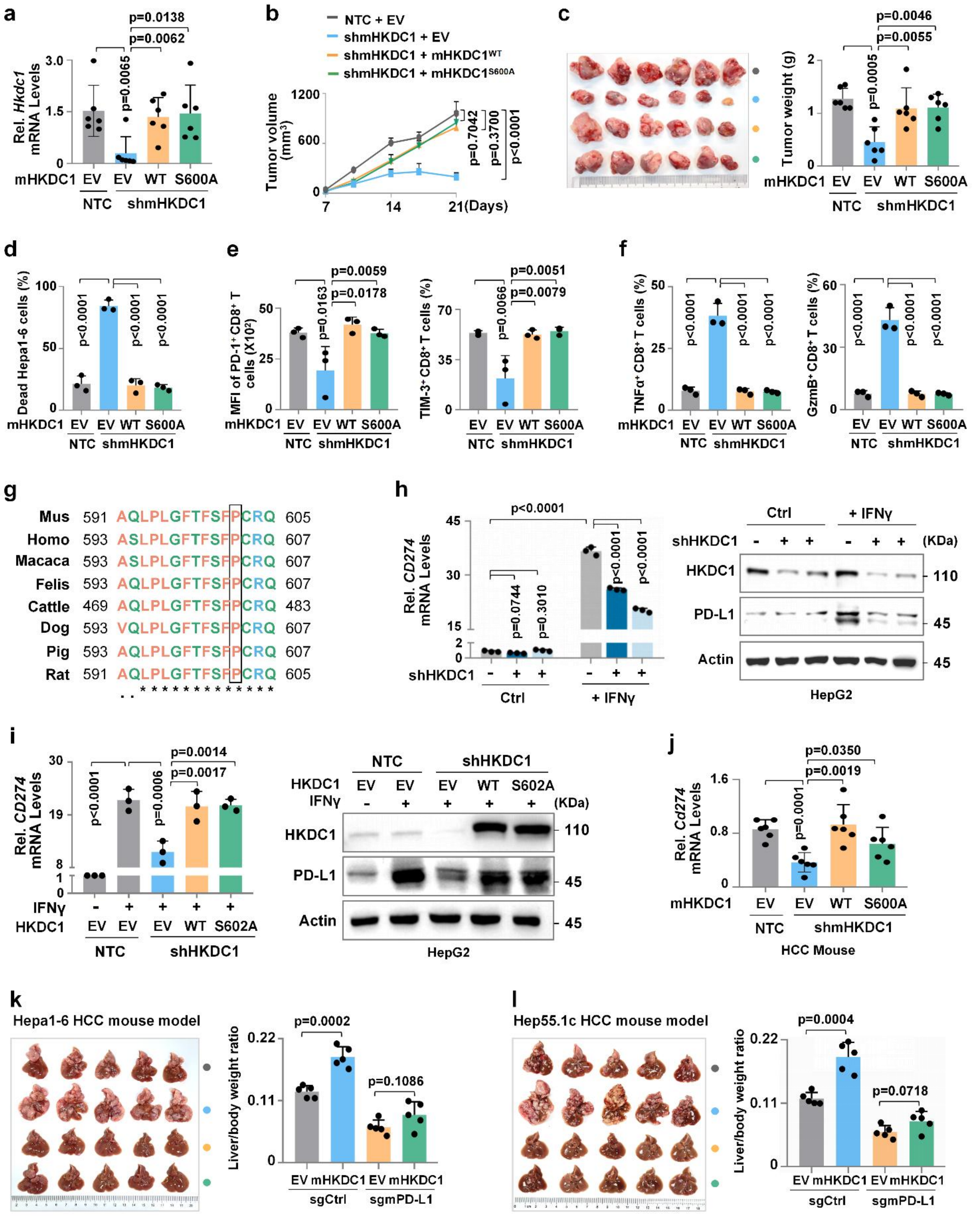

Supplementary Fig. 3 | HKDC1 enhances PD-L1-mediated immune evasion of HCC cells independent of its hexokinase activity. Related to Fig. 2.

a, qPCR analysis of mRNA levels of *hkdc1* in the hepatic portal mouse model in (**2c**). **b**, Tumor growth analysis of Hepa1-6 xenografts with indicated genotypes. Hepa1-6 cells were injected subcutaneously into C57BL/6 mice (6-8 weeks, n = 6 male mice per group). **c**, Tumor weight of Hepa1-6 xenografts in (**b**) was measured at the end of the experiment. **d**, Cell death analysis of indicated Hepa1-6 cells co-cultured with activated CD8$^+$ T cells was analyzed by flow cytometry. **e**,

The median fluorescence intensity (MFI) of PD-1^+ and the percentage of TIM-3^+ $CD8^+$ T cells co-cultured with Hepa1-6 cells with indicated genotypes was analyzed by flow cytometry. **f**, The percentage of $TNF\alpha^+$ or $GzmB^+$ $CD8^+$ T cells co-cultured with Hepa1-6 cells with indicated genotypes was analyzed by flow cytometry. **g**, Alignment of HKDC1 protein homologous sequences from different species. **h**, qPCR analysis and western blot analysis of PD-L1 in HepG2 cells with indicated genotypes with or without IFNγ stimulation. **i**, qPCR analysis and western blot analysis of PD-L1 in HepG2 cells with indicated genotypes with or without IFNγ stimulation. **j**, qPCR analysis of *Cd274* in the hepatic portal mouse model in (**2c**). **k**, Image of livers in Hepa1-6 xenografts is shown on the left. Hepa1-6 cells with indicated genotypes were injected through hepatic portal vein into C57BL/6 mice (6-8 weeks, n = 5 mice per group). Compared liver/body weight ratio is shown on the right. **l**, Image of livers in Hep55.1c xenografts is shown on the left. Hep55.1c cells with indicated genotypes were injected through hepatic portal vein into C57BL/6 mice (6-8 weeks, n = 5 mice per group). Compared liver/body weight ratio is shown on the right. Data are presented as mean ± SD (**a**-**c**, **j**-**l**). Data are presented as mean ± s.e.m of three biologically independent experiments (**d**-**f**, **h**, **i**). Representative blot shown from three biologically independent experiments (**h**, **i**). P-values were calculated by two-tailed unpaired Student's t-test (**a**, **j**-**l**), one-way ANOVA (**c**-**f**, **h**, **i**) or two-way ANOVA (**b**). Source data are provided as a Source Data file.

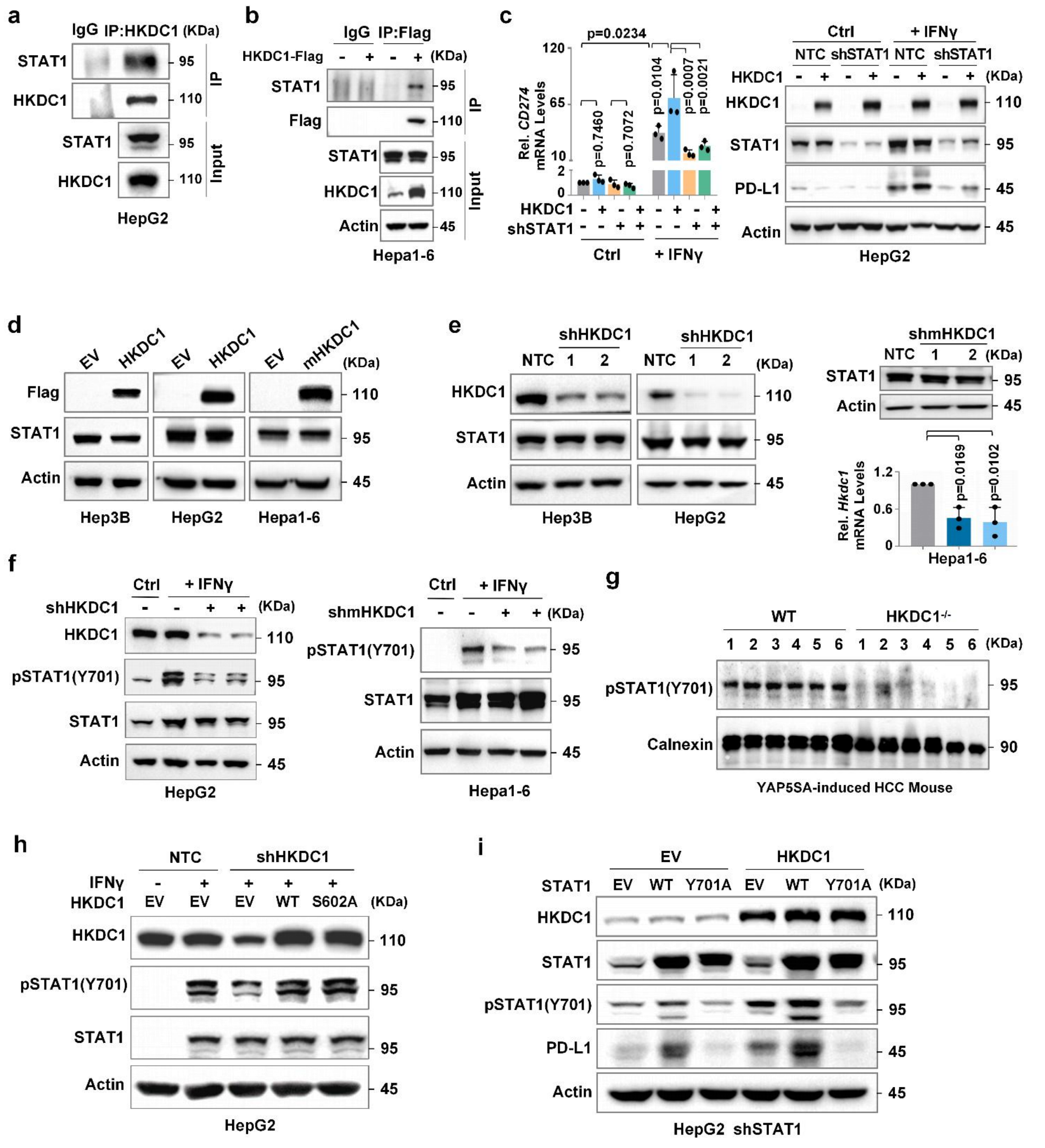

Supplementary Fig. 4 | HKDC1 associates with STAT1 and facilitates its phosphorylation to enhance PD-L1 expression. Related to Fig. 3.

a, Immunoprecipitation assay of interaction between endogenous HKDC1 and STAT1 in HepG2 cells. The lysates were immunoprecipitated with HKDC1 antibody, followed by Western blot analysis with antibodies against HKDC1 and STAT1. **b**, Immunoprecipitation assay of interaction between HKDC1 and STAT1. Hepa1-6 cells were transfected with Flag-tagged HKDC1 plasmid. The lysates were immunoprecipitated with an anti-Flag antibody, followed by Western blot analysis with antibodies against Flag-tag and STAT1. **c**, qPCR analysis and Western blot analysis of the levels of PD-L1 in HepG2 cells with indicated genotypes with or without IFNγ stimulation. **d**-**e**, Western blot analysis of protein levels of STAT1 in Hep3B, HepG2 and Hepa1-6 cells with indicated genotypes. **f**, Western blot analysis of protein levels of STAT1 and Tyr701 phosphorylated

STAT1 in HepG2 and Hepa1-6 cells expressing NTC or shHKDC1 with or without IFNγ stimulation. **g**, Western blot analysis of protein levels of HKDC1 and Tyr701 phosphorylated STAT1 in tumor tissues of YAP5SA-induced HCC mouse model with indicated genotypes (*n* = 6 male mice per group). **h**, Western blot analysis of protein levels of STAT1 and Tyr701 phosphorylated STAT1 in HepG2 cells with indicated genotypes with or without IFNγ stimulation. **i**, Western blot analysis of protein levels of PD-L1, STAT1 and Tyr701 phosphorylated STAT1 in IFNγ-stimulated endogenous STAT1-knockdown Hep3B cells with indicated phenotypes. Representative blot shown from three biologically independent experiments (**a**-**i**). Data are presented as mean ± s.e.m of three biologically independent experiments (**c**, **e**). *P*-values were calculated by one-way ANOVA (**c**, **e**). Source data are provided as a Source Data file.

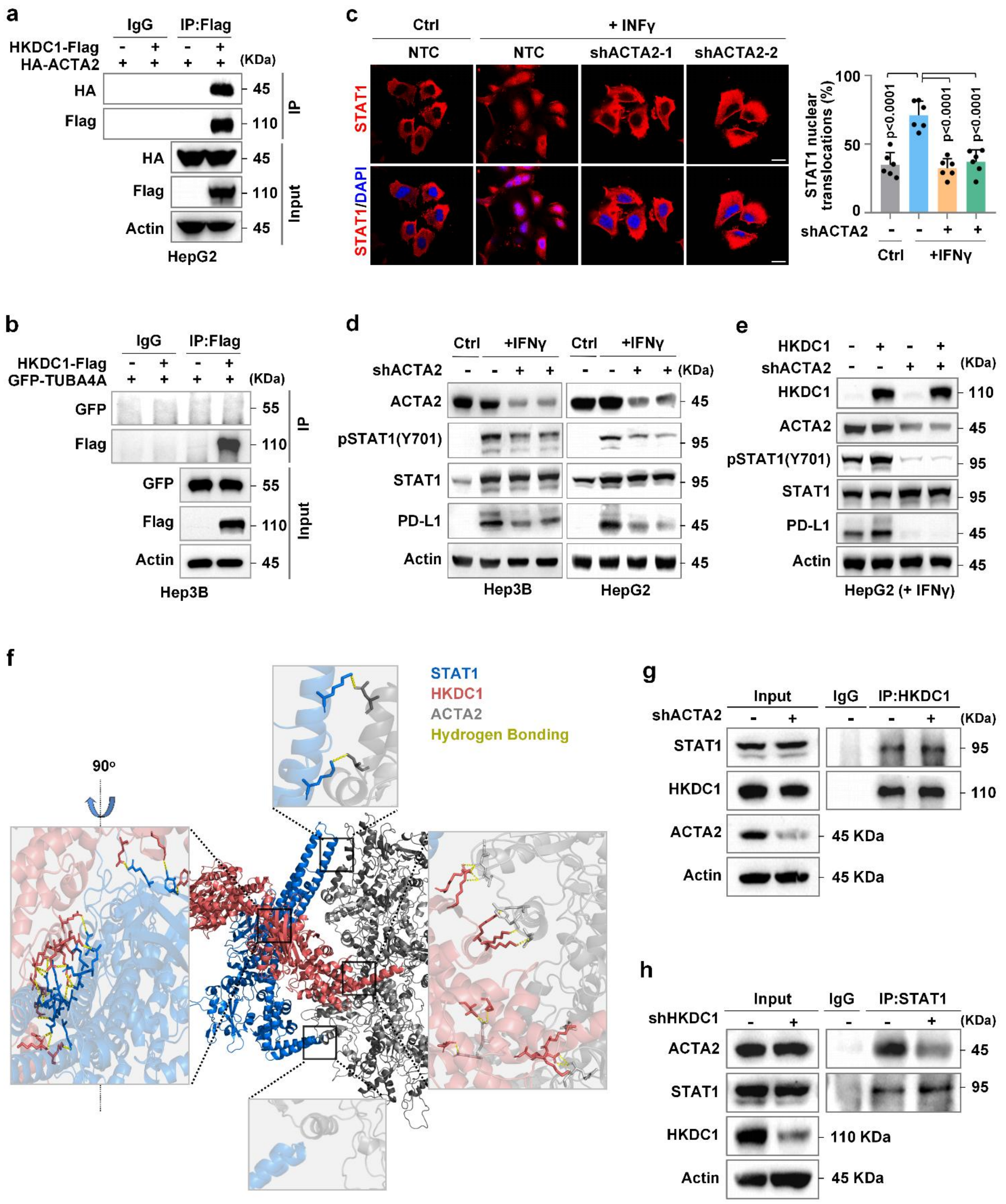

Supplementary Fig. 5 | HKDC1 presents cytosolic STAT1 to IFNGR1 to facilitate its phosphorylation via actin cytoskeleton protein ACTA2. Related to Fig. 4.

a, Immunoprecipitation assay of interaction between HKDC1 and ACTA2. HepG2 cells were transfected with HA-tagged ACTA2 and Flag-tagged HKDC1 plasmids. **b**, Immunoprecipitation assay of interaction between HKDC1 and TUBA4A. Hep3B cells were transfected with Flag-tagged HKDC1 and GFP-tagged TUBA4A plasmids. **c**, Representative immunofluorescence staining for STAT1 in Hep3B cells expressing NTC or shACTA2 with or without IFNγ stimulation. The nucleus was stained with DAPI. The percentage of nuclear STAT1 (right) in each group was quantitated using ImageJ. Scale bars, 20 μm. **d**, Western blot analysis of protein levels of PD-L1, STAT1 and

Tyr701 phosphorylated STAT1 in Hep3B and HepG2 cells expressing NTC or shACTA2 with or without IFNγ stimulation. **e**, Western blot analysis of protein levels of PD-L1, STAT1 and Tyr701 phosphorylated STAT1 in IFNγ-stimulated endogenous HKDC1-knockdown HepG2 cells with indicated genotypes. **f**, Protein-structures of HKDC1 (red) and STAT1 (blue) were predicted by AlphaFold2, and protein-structure of ACTA2 (grey) is referred to known structure. Protein structure of HKDC1, STAT1 and ACTA2 were docked with HDOCK. Short yellow bonds represent hydrogen bonds. **g**, Immunoprecipitation assay of interaction between endogenous HKDC1 and STAT1 in Hep3B cells expressing NTC or shACTA2. **h**, Immunoprecipitation assay of interaction between endogenous ACTA2 and STAT1 in Hep3B cells expressing NTC or shHKDC1. Representative blot and Immunofluorescence image shown from three biologically independent experiments (**a**-–**e, g, h**). Data are presented as mean ± s.e.m of six biologically independent experiments (**c**). *P*-values were calculated by one-way ANOVA (**c**). Source data are provided as a Source Data file.

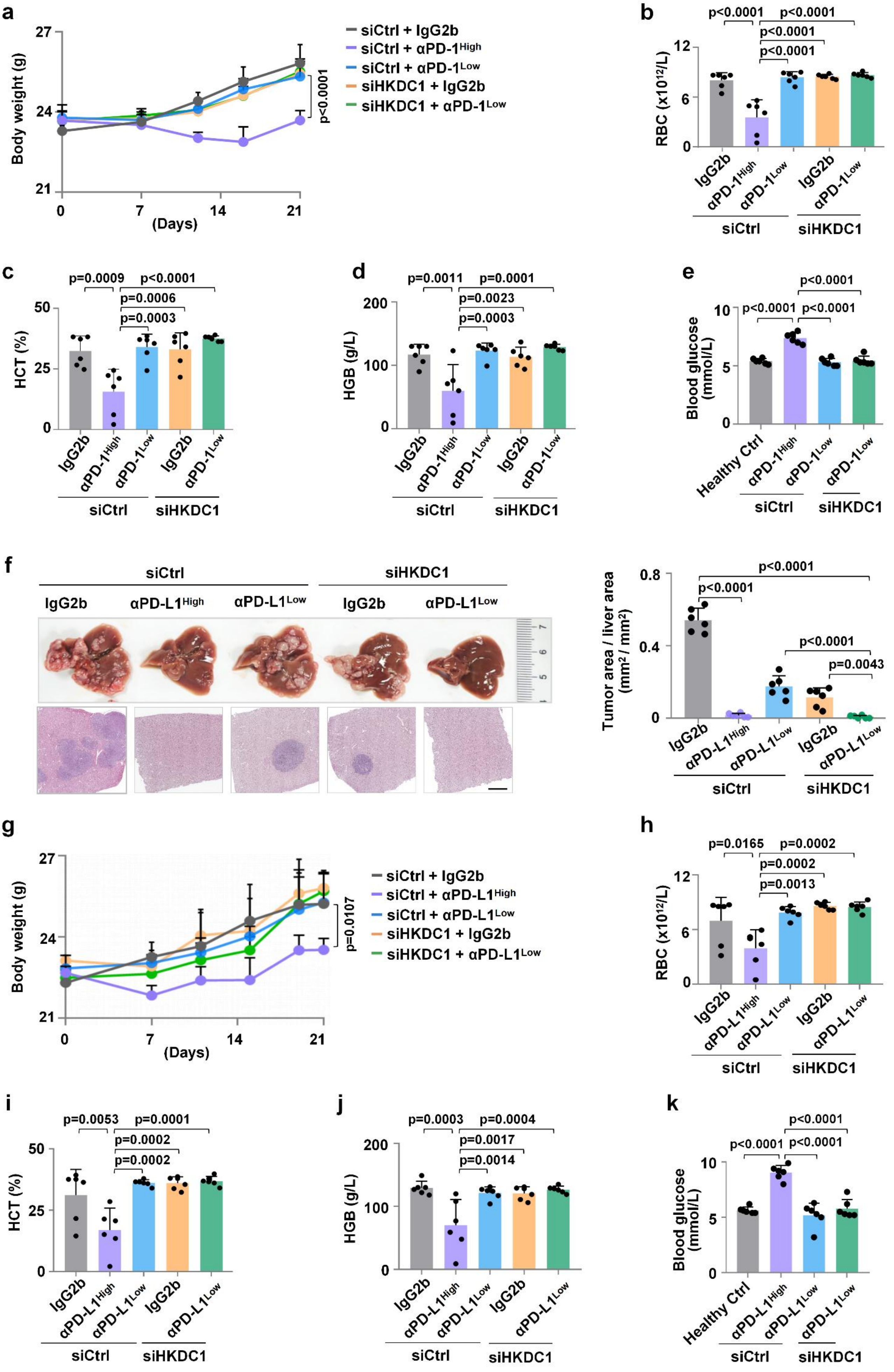
a
Body weight (g)
27
24
21
0
7
14
21
(Days)
p<0.0001
siCtrl + IgG2b
siCtrl + αPD-1High
siCtrl + αPD-1Low
siHKDC1 + IgG2b
siHKDC1 + αPD-1Low
b
RBC (x10^12/L)
p<0.0001
p<0.0001
p<0.0001
p<0.0001
IgG2b
αPD-1High
αPD-1Low
IgG2b
αPD-1Low
siCtrl
siHKDC1
c
HCT (%)
p=0.0009
p<0.0001
p=0.0006
p=0.0003
d
HGB (g/L)
p=0.0011
p=0.0001
p=0.0023
p=0.0003
e
Blood glucose (mmol/L)
p<0.0001
p<0.0001
p<0.0001
Healthy Ctrl
f
siCtrl
siHKDC1
IgG2b
αPD-L1High
αPD-L1Low
IgG2b
αPD-L1Low
Tumor area / liver area (mm² / mm²)
p<0.0001
p<0.0001
p<0.0001
p=0.0043
g
Body weight (g)
(Days)
p=0.0107
siCtrl + IgG2b
siCtrl + αPD-L1High
siCtrl + αPD-L1Low
siHKDC1 + IgG2b
siHKDC1 + αPD-L1Low
h
RBC (x10^12/L)
p=0.0165
p=0.0002
p=0.0002
p=0.0013
i
HCT (%)
p=0.0053
p=0.0001
p=0.0002
p=0.0002
j
HGB (g/L)
p=0.0003
p=0.0004
p=0.0017
p=0.0014
k
Blood glucose (mmol/L)
p<0.0001
p<0.0001
p<0.0001
Healthy Ctrl

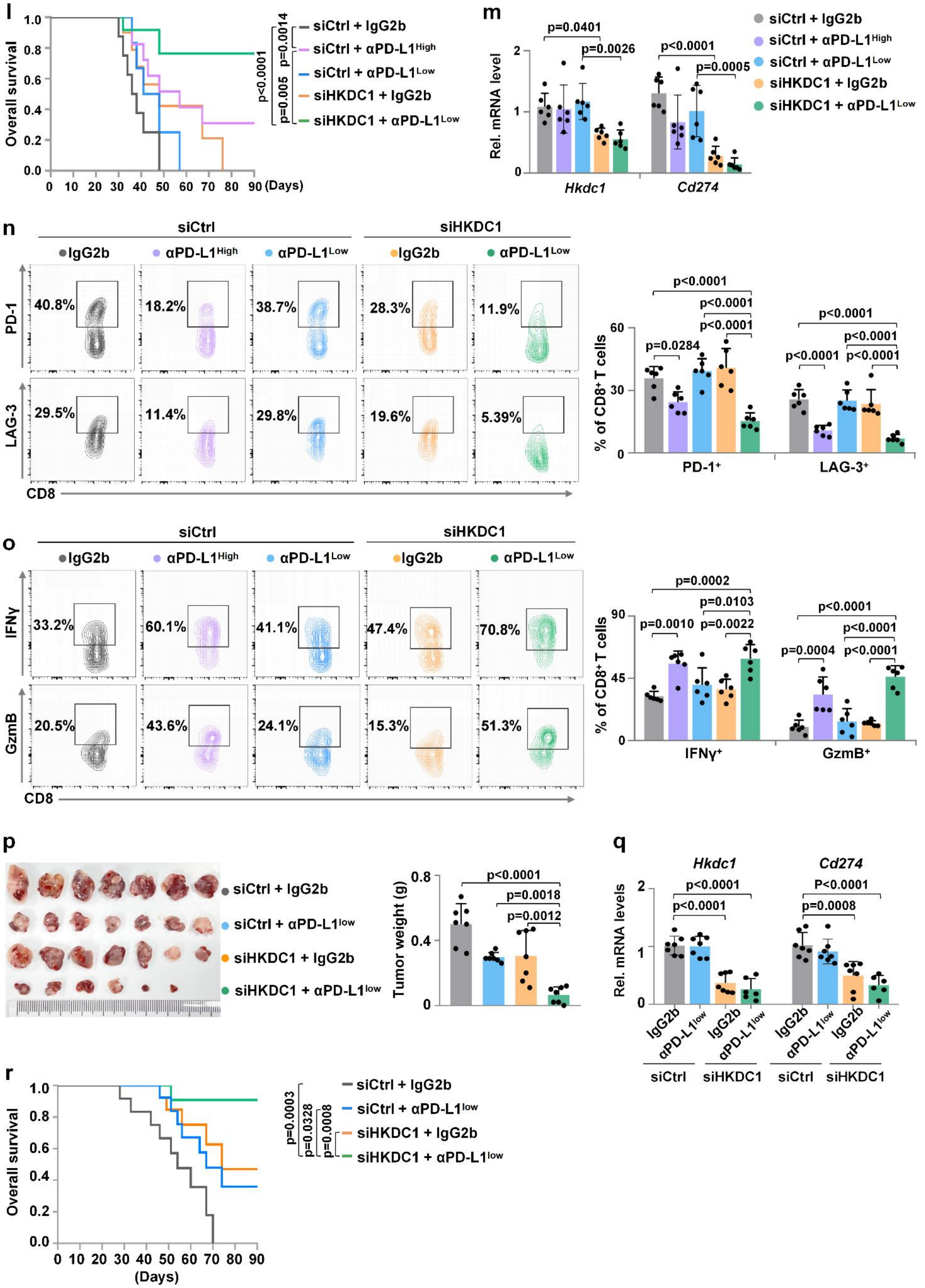

Supplementary Fig. 6 | HKDC1 inhibition incorporating with PD-1/PD-L1 blockade enhances T cell antitumor response in HCC model mice. Related to Fig. 5.

a, Body weight of hepatic portal mouse model in **(Fig. 5a)**. **b-d**, RBC, HCT and HGB in peripheral

blood of hepatic portal mouse model in (**Fig. 5a**) were measured before sacrifice. **e**, Levels of blood glucose of hepatic portal mouse model in (**Fig. 5a**) were measured before sacrifice. **f**, The H.E. quantification of tumor area/liver area (mm^2/mm^2) in hepatic portal mouse model with indicated genotypes (n = 6 male mice per group) is displayed on the right. Hepa1-6 cells were injected through hepatic portal vein into C57BL/6 mice (6-8 weeks), and randomly divided into five groups. $VNP^{Ctrl/MTO}$ or $VNP^{siHKDC1/MTO}$ (0.34 OD/per mice) was injected through tail vein, then αPD-L1 or IgG2b isotype control was injected intraperitoneally twice a week. Images of liver and representative H.E. staining are displayed on the left. Scale bars, 500 μm. **g**, Body weight of hepatic portal mouse model in (**f**). **h-j**, RBC, HCT and HGB in peripheral blood of hepatic portal mouse model in (**f**) were measured before sacrifice. **k**, Levels of blood glucose of hepatic portal mouse model in (**f**) were measured before sacrifice. **l**, Overall survival of hepatic portal mouse model in (**f**). **m**, mRNA levels of *Hkdc1* and *Cd274* in the hepatic portal mouse model in (**f**). **n**, The percentage of tumor-infiltrating $PD\text{-}1^+$ or $LAG\text{-}3^+$ $CD8^+$ T cells in hepatic portal mouse model in (**f**) was analyzed by flow cytometry. **o**, The percentage of tumor-infiltrating $IFN\gamma^+$ or $GzmB^+$ $CD8^+$ T cells in hepatic portal mouse model in (**f**) was analyzed by flow cytometry. **p**, Tumor image and tumor weight of Hepa1-6 xenografts with indicated treatment (n = 7 male mice per group). Hepa1-6 cells were injected subcutaneously into C57BL/6 mice (6-8 weeks), and randomly divided into four groups. $VNP^{Ctrl/MTO}$ or $VNP^{siHKDC1/MTO}$ (0.34 OD/per mice) was injected through tail vain and αPD-L1 or IgG2b isotype control was injected intraperitoneally twice a week. **q**, mRNA levels of *Hkdc1* and *Cd274* in the Hepa1-6 xenografts in (**p**). **r**, Overall survival of Hepa1-6 xenografts mice in (**p**). Data are presented as mean ± SD (**a**-**r**). *P*-values were calculated by one-way ANOVA (**b**-**k, m-q**), two-way ANOVA (**a,g**) or log-rank (Mantel-Cox) test (**l,r**). Source data are provided as a Source Data file.

a

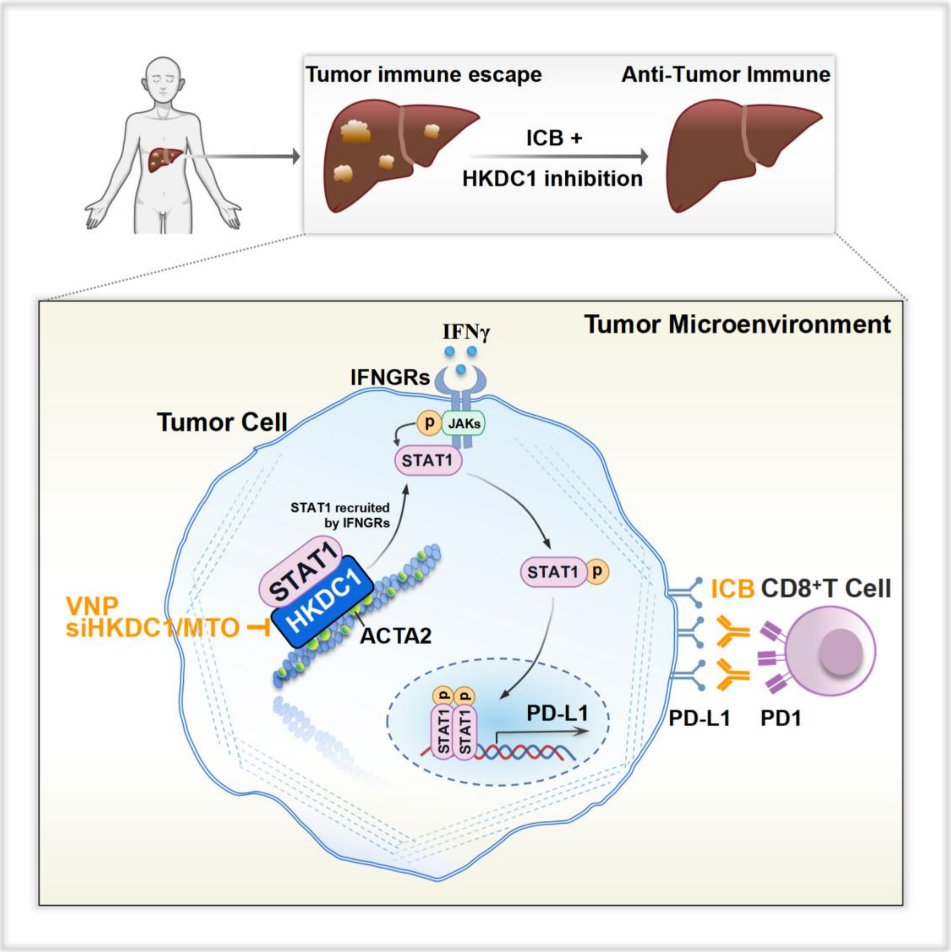

Supplementary Fig. 7 | HKDC1 promotes tumor immune evasion in a CD8^{+} T cell-dependent manner by coupling cytoskeleton to STAT1/PD-L1 activation. a, HKDC1 binds to and presents cytosolic STAT1 to IFNGR1 on the plasma membrane following IFNγ-stimulation by associating with cytoskeleton protein ACTA2, resulting in STAT1 phosphorylation and nuclear translocation, and upregulation of PD-L1 expression. HKDC1 inhibition incorporating with anti-PD-1/PD-L1 enhances T cell antitumor response.

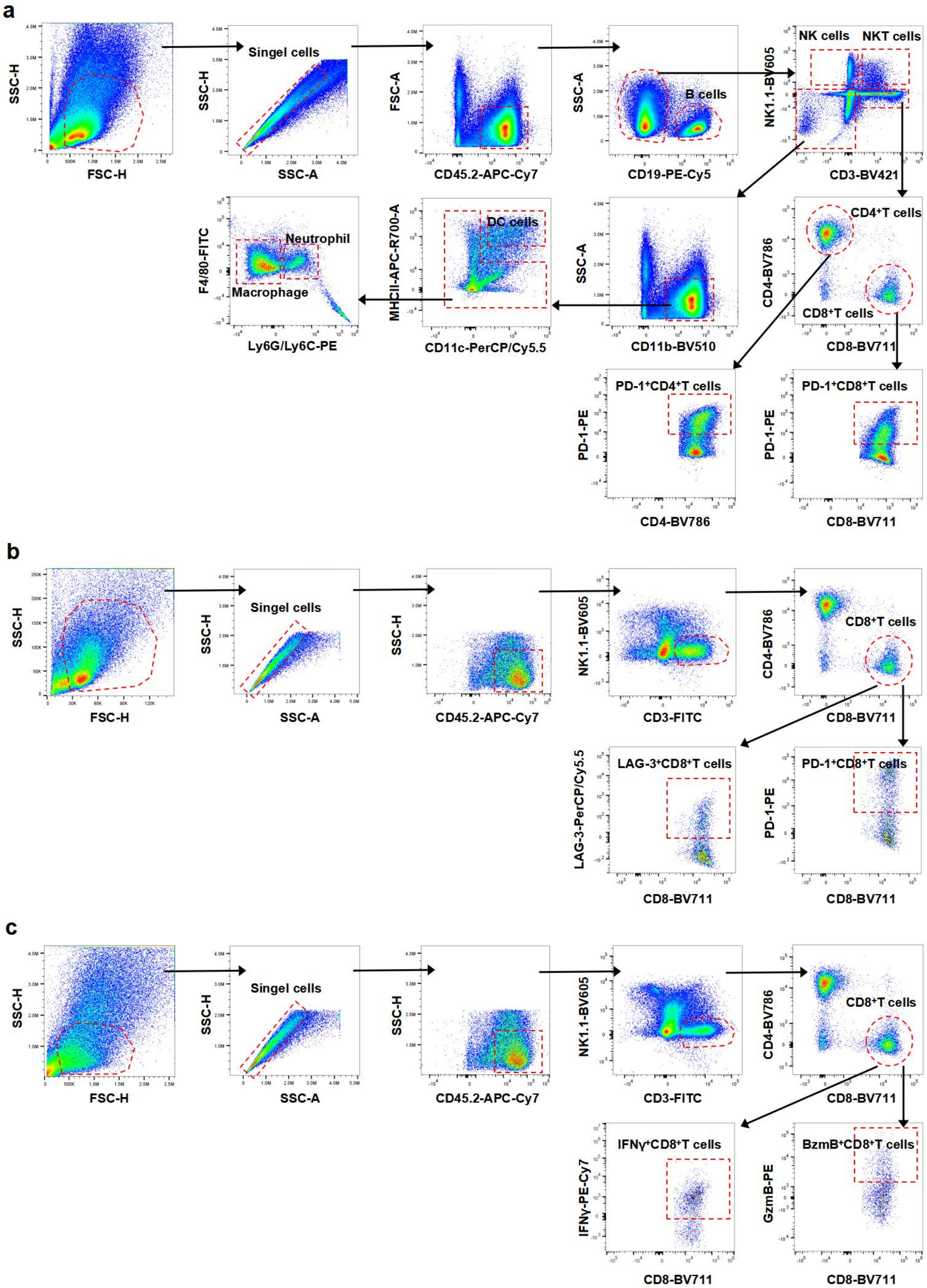
a
SSC-H
FSC-H
Singel cells
SSC-A
FSC-A
CD45.2-APC-Cy7
B cells
CD19-PE-Cy5
NK cells
NKT cells
NK1.1-BV605
CD3-BV421
F4/80-FITC
Neutrophil
Macrophage
Ly6G/Ly6C-PE
MHCII-APC-R700-A
DC cells
CD11c-PerCP/Cy5.5
CD11b-BV510
CD4+T cells
CD4-BV786
CD8+T cells
CD8-BV711
PD-1+CD4+T cells
PD-1-PE
PD-1+CD8+T cells
b
SSC-H
FSC-H
Singel cells
SSC-A
CD45.2-APC-Cy7
NK1.1-BV605
CD3-FITC
CD4-BV786
CD8+T cells
CD8-BV711
LAG-3+CD8+T cells
LAG-3-PerCP/Cy5.5
PD-1+CD8+T cells
PD-1-PE
c
SSC-H
FSC-H
Singel cells
SSC-A
CD45.2-APC-Cy7
NK1.1-BV605
CD3-FITC
CD4-BV786
CD8+T cells
CD8-BV711
IFNγ+CD8+T cells
IFNγ-PE-Cy7
BzmB+CD8+T cells
GzmB-PE

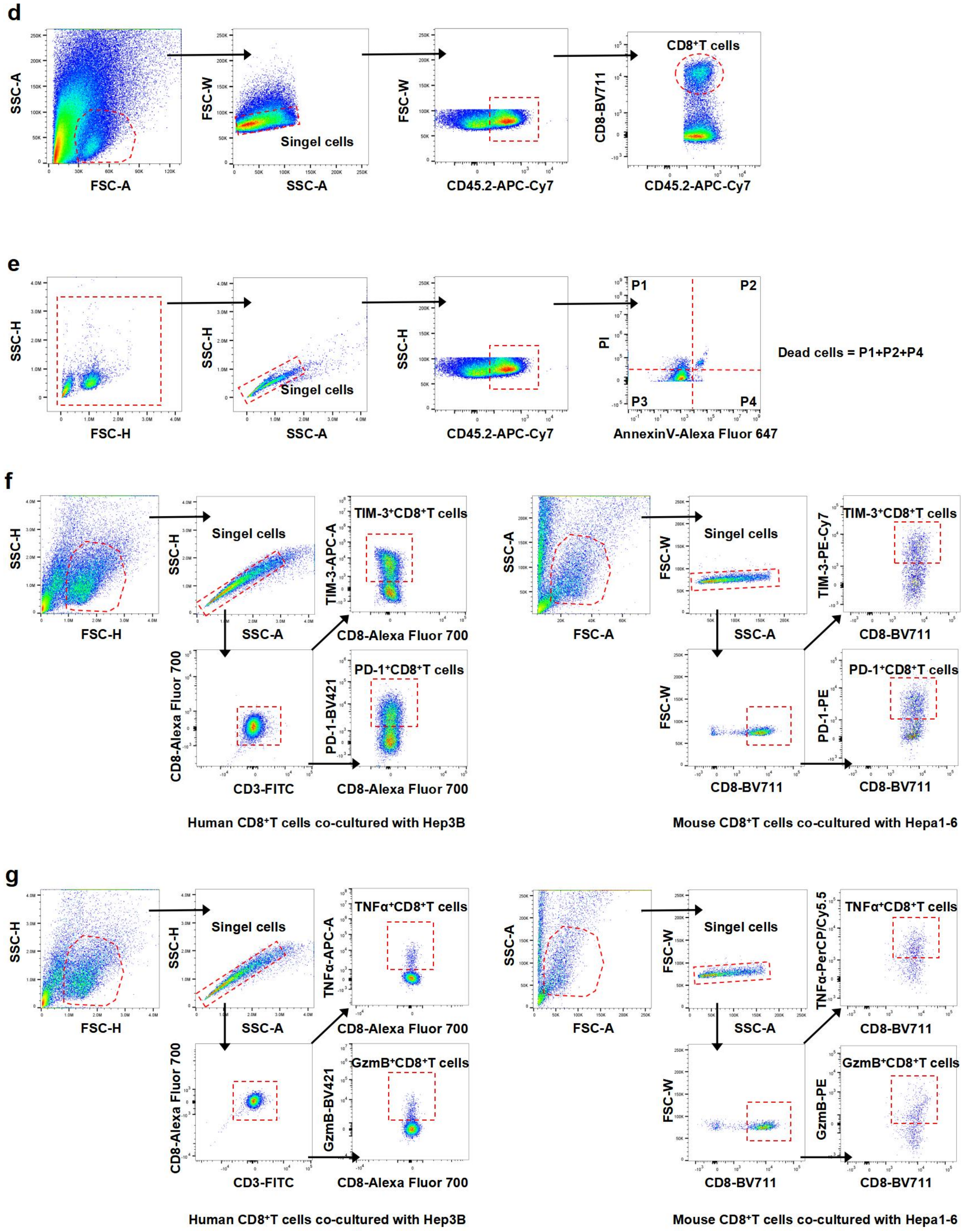

Supplementary Fig. 8 | Gating and sorting strategies. a, Representative flow cytometry gating and sorting strategies of tumor-infiltrating immune cells in HCC mouse model (Fig. 1b and Supplementary Fig. 2a). **b**, Representative flow cytometry gating strategies of PD-1^{+} CD8^{+} T cells and LAG-3^{+} CD8^{+} T cells in HCC mouse model (Fig. 1c, Fig. 2d, Fig. 5d, Supplementary Fig. 2c, Supplementary Fig. 2f and Supplementary Fig. 6n). **c**, Representative flow cytometry gating strategies of IFNγ^{+} CD8^{+} T cells and GzmB^{+} CD8^{+} T cells in HCC mouse model (Fig. 1d, Fig. 2e,

Fig. 5e, Supplementary Fig. 2d, Supplementary Fig. 2g and Supplementary Fig. 6o). **d**, Representative flow cytometry gating strategies of $CD8^+$ T cells in HCC mouse model (Fig. 1f). **e**, Representative flow cytometry gating strategies of dead Hep3B or Hepa1-6 cells co-cultured with $CD8^+$ T cells (Fig. 1g, Fig. 2f, Fig. 4j, Supplementary Fig. 2h and Supplementary Fig. 3d). **f**, Representative flow cytometry gating strategies of PD-1^+ $CD8^+$ T cells and TIM-3^+ $CD8^+$ T cells co-cultured with Hep3B or Hepa1-6 cells (Fig. 1h, Fig. 2g, Fig. 4k, Supplementary Fig. 2i and Supplementary Fig. 3e). **g**, Representative flow cytometry gating strategies of $TNF\alpha^+$ $CD8^+$ T cells and $GzmB^+$ $CD8^+$ T cells co-cultured with Hep3B or Hepa1-6 cells (Fig. 1i, Fig. 2h, Fig. 4l, Supplementary Fig. 2i and Supplementary Fig. 3f).

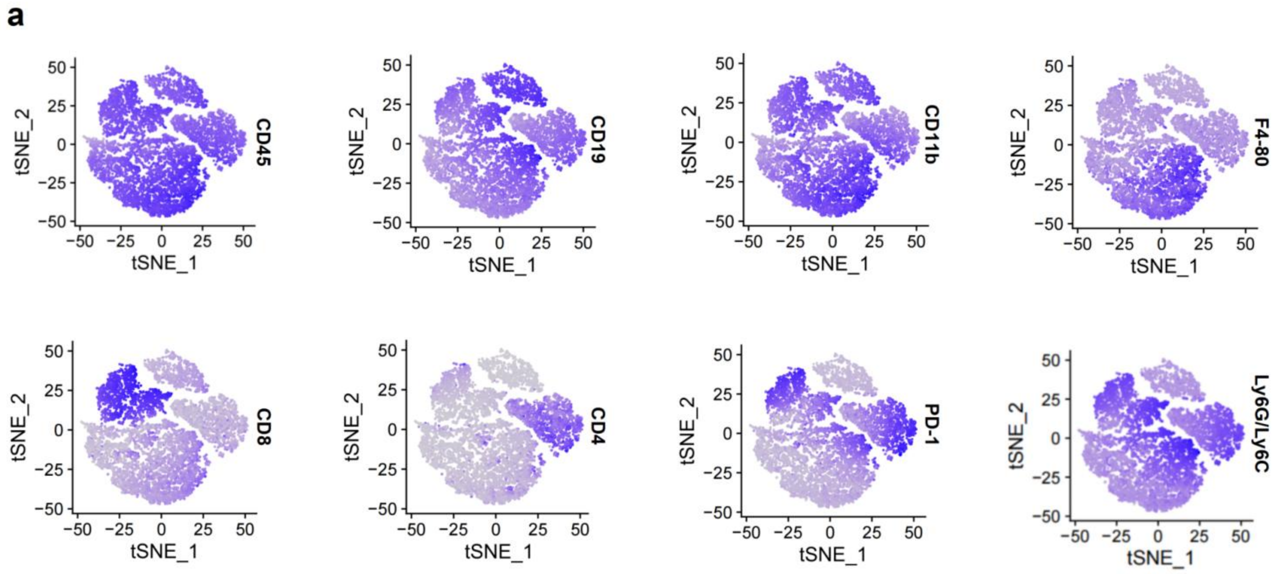

Supplementary Fig. 9 | Individual analysis of t-SNE. a, t-SNE plot shows the projection of various immune cells. The functional description of each cluster shown in different colors is determined by the gene expression characteristics of each cluster (Fig. 1b).

Supplementary Table 1. Targeting Sequence of shRNAs.

Species	Name	Targeted Sequence
Homo sapiens	HKDC1-sh1	5’-GCTGCGGGACTTGGAAATATA-3’
	HKDC1-sh2	5’-GCGGGACTTGGAAATATATAG-3’
Mus musculus	mHKDC1-sh1	5’-CAATGAAATCACCCGTGGGAA-3’
	mHKDC1-sh2	5’-GCACCTCCATTTGGCATATTT-3’
Mus musculus	simHKDC1	5’-GUGACAUUUAUGUUGUCAGAA-3’
Mus musculus	sgmPD-L1	5’-GACGTCAAGCTGCAGGACGC-3’
Homo sapiens	STAT1-sh1	5’-GAACAGAAATACACCTACGAA-3’
	STAT1-sh2	5’-CGACAGTATGATGAACACAGT-3’
Homo sapiens	ACTA2-sh1	5’-GAGATGGTGTCACCCACAATG-3’
	ACTA2-sh2	5’-GCTATTCCTTCGTTACTACTG-3’

Supplementary Table 2. Oligonucleotide primers used for qPCR.

Name	Species	Accession	Primer Sequences
HKDC1	*Homo sapiens*	NM_025130.4	TGAGCCGTCTGACCAAAGC
			TAGGGGTCGTCATAGGCACA
STAT1	*Homo sapiens*	NM_001384880.1	TACGGAAAAGCAAGCGTAATCT
			TGCACATGACTTGATCCTTCAC
CD274	*Homo sapiens*	NM_014143.4	TGCCGACTACAAGCGAATTACTG
			CTGCTTGTCCAGATGACTTCGG
GAL9	*Homo sapiens*	NM_009587.3	ACACCCAGATCGACAACTCCTG
			CAAACAGGTGCTGACCATCCAC
CD276	*Homo sapiens*	NM_001024736.2	CTGGCTTTCGTGTGCTGGAGAA
			GCTGTCAGAGTGTTTCAGAGGC
CD133	*Homo sapiens*	NM_006017.3	AGTCGGAAACTGGCAGATAGC
			GGTAGTGTTGTACTGGGCCAAT
EpCAM	*Homo sapiens*	NM_002354.3	ATAACCTGCTCTGAGCGAGTG
			TGCAGTCCGCAAACTTTTACTA
GPC3	*Homo sapiens*	NM_001164617.2	ATTGGCAAGTTATGTGCCCAT
			TTCGGCTGGATAAGGTTTCTTC
MUC1	*Homo sapiens*	NM_002456.6	CCAGTTCAAGCGACCCTTTTA
			CGCTGTGGGATACTGTTGATT
CEACAM18	*Homo sapiens*	NM_001405061.1	GCCTACCTCTAGTAGTGACCG
			CTGGGAAACTCTCTATCATGCAC
CEACAM4	*Homo sapiens*	NM_011964.2	ATTCAAGCAAATATCCCAGGGG
			GGCATTTATGGTTCGTAGGGTG
CD86	*Homo sapiens*	NM_175862.5	CTGCTCATCTATACACGGTTACC
			GGAAACGTCGTACAGTTCTGTG
CD80	*Homo sapiens*	NM_005191.4	GGCCCGAGTACAAGAACCG
			TCGTATGTGCCCTCGTCAGAT
VSIR	*Homo sapiens*	NM_022153.2	ACGCCGTATTCCCTGTATGTC
			TTGTAGAAGGTCACATCGTGC
PDCD1LG2	*Homo sapiens*	NM_025239.4	ACCCTGGAATGCAACTTTGAC
			AAGTGGCTCTTTCACGGTGTG
VTCN1	*Homo sapiens*	NM_024626.4	CTCACAGATGCTGGCACCTACA
			GCAAGGTCTCTGAGCTGGCATT
UNQ329	*Homo sapiens*	NM_003820.4	GTGCAGTCCAGGTTATCGTGT
			CACTTGCTTAGGCCATTGAGG
TNFSF9	*Homo sapiens*	NM_003811.4	GGCTGGAGTCTACTATGTCTTCT

			ACCTCGGTGAAGGGAGTCC
TNFSF4	*Homo sapiens*	NM_003326.5	CCAGGCCAAGATTCGAGAGG
			CCGATGTGATACCTGAAGAGCA
CD70	*Homo sapiens*	NM_001252.5	GCTTTGGTCCCATTGGTCG
			CGTCCCACCCAAGTGACTC
CD40	*Homo sapiens*	NM_001250.6	ACATACAACCAAACTTCTCCCCG
			GCAAAAAGTGCTGACCCAATCA
PVR	*Homo sapiens*	NM_006505.5	TGGAGGTGACGCATGTGTC
			GTTTGGACTCCGAATAGCTGG
CD226	*Homo sapiens*	NM_006566.4	ATAGCCACATTGTTTCGGAACC
			ATCTGACGGGGCTGGATCTTT
18S	*Homo sapiens*		CGGCGACGACCCATTCGAAC
			GAATCGAACCCTGATTCCCCGTC
Hkdc1	*Mus musculus*	XM_006513473.4	ACACTTGGTGGCGTTTTACTT
			CCGCATGTGATACAGGAACC
Stat1	*Mus musculus*	NM_001205314	TCACAGTGGTTCGAGCTTCAG
			CGAGACATCATAGGCAGCGTG
Cd274	*Mus musculus*	NM_021893	GCTCCAAAGGACTTGTACGTG
			TGATCTGAAGGGCAGCATTTC
Gapdh	*Mus musculus*	NM_008084	TGTGTCCGTCGTGGATCTGA
			TTGCTGTTGAAGTCGCAGGAG
Mhc1	*Mus musculus*	NM_001025208.1	GAGGGTGGCTCTCACACATTC
			TTGGCCTTCGTAAGCAAACTG
Pdcd1lg2	*Mus musculus*	NM_021396.2	GCTCCAAAGGACTTGTACGTG
			TGATCTGAAGGGCAGCATTTC
Gal9	*Mus musculus*	NM_010708.2	CTGCCGATACTGAACCTGAGC
			GCGGTCAAAATCGCACTCC
Cd276	*Mus musculus*	NM_133983.4	TTACTGGACCAATCCAAGGAGG
			AGCTGTTCTGAAAGTTCACCAC
Vtcn1	*Mus musculus*	NM_178594.4	CACCCTACGCTGCTCCTTTTC
			CCCTCGTCGGTTACTCGGA
Cd133	*Mus musculus*	NM_001163577.1	CATCGCACTCATCATTGGCTT
			TTGAACTCGTGGACCAAACCT
Gpc3	*Mus musculus*	NM_016697.3	ACTGGGGCTGTGTGGAAAG
			GCATTGAAGGTATCTTGGGTCTC
Muc1	*Mus musculus*	NM_013605.2	ACCTGGCTAATGACTAGCTCA
			AGACCTGTAAAGGATTTGCGTC

Psg19	*Mus musculus*	NM_011964.2	TGCTGTGACGCTTTCAACTCT
			GGCCACTGATGATAGACTCTGTC
Ceacam9	*Mus musculus*	NM_011927.4	TTAACCTGCTGGAATGCACCC
			CAGAACGGAGTTTCCGCCTT
Cd80	*Mus musculus*	NM_001359898.1	TCAGTTGATGCAGGATACACCA
			AAAGACGAATCAGCAGCACAA
Cd86	*Mus musculus*	NM_019388.3	TCAATGGGACTGCATATCTGCC
			GCCAAAATACTACCAGCTCACT
Unq329	*Mus musculus*	NM_178931.2	ACTGCATCAACGTCTTGGAGA
			TGGCTCCTGTAAGATGTGCTG
Tnfsf9	*Mus musculus*	NM_009404.3	CGGCGCTCCTCAGAGATAC
			ATCCCGAACATTAACCGCAGG
Cd70	*Mus musculus*	NM_011617.2	TGTAGCGGACTACTCAGTAAGC
			GGGGTCCTTCCGAGGAACT
Cd40	*Mus musculus*	NM_011611.2	TGTCATCTGTGAAAAGGTGGTC
			ACTGGAGCAGCGGTGTTATG
Cd48	*Mus musculus*	NM_007649.5	ACGAGTTGAAGATAACCCTGGA
			CACGATAGCCTCAGGTGACAG
Nectin4	*Mus musculus*	NM_027893.3	CCCTGCTTATGAGGACCGC
			CTGACTCTGCATTCGTACTCG
Cd69	*Mus musculus*	NM_001033122.4	CCCTTGGGCTGTGTTAATAGTG
			AACTTCTCGTACAAGCCTGGG
Cd226	*Mus musculus*	NM_001039149.1	CCATCGTTGGAGGGTTAGTTTC
			CCCTGGGCTCTTTAAGTGGAA

Supplementary Table 3. Reagents.

Reagents	Source	Identifier
HKDC1 Polyclonal, 1:1000	Proteintech	cat: 25874-1-AP
STAT1 Polyclonal, 1:3000	Proteintech	cat: 10144-2-AP
STAT1 Monoclonal, 1:1000	Abcam	cat: AB234400
PD-L1 Polyclonal, 1:3000	Proteintech	cat: 28076-1-AP
STAT1 (Phospho-Tyr701) Polyclonal, 1:500	Sangon Biotech	cat: D155017
STAT1 (Phospho-Ser727) Monoclonal (clone: EPR3146), 1:500	Abcam	cat: ab109461
ACTA2 Monoclonal (clone: EPR5368), 1:15000	Abcam	cat: ab124964
β-Actin Monoclonal, 1:5000	Proteintech	cat: 66009-1-Ig
Calnexin Polyclonal, 1:5000	Proteintech	cat: 10427-2-AP
LaminB Polyclonal, 1:5000	Proteintech	cat: 12987-1-AP
α-Tubulin Monoclonal, 1:3000	Proteintech	cat: 66031-1-Ig
HRP-conjugated anti-rabbit, 1:10000	Bio-Rad	cat: 170-6515
HRP-conjugated anti-mouse, 1:10000	Bio-Rad	cat: 170-6516
HA-Tag Monoclonal, 1:2000	Proteintech	cat: 66006-2-Ig
DYKDDDDK-tag Monoclonal, 1:2000	Proteintech	cat: 66008-3-Ig
GFP-tag Polyclonal, 1:3000	Proteintech	cat: 50430-2-AP
GST-tag Monoclonal,1:3000	Proteintech	cat: 66001-2-Ig
His-Tag Monoclonal, 1:5000	Proteintech	cat: 66005-1-Ig
Flag-M2 Monoclonal (clone: M2), 1:1000	Sigma-Aldrich	cat: F1804
Ms TNF BB700 (clone: MP6-XT22), 1:100	BD Biosciences	cat: 566510
PerCP/Cyanine5.5 anti-mouse TNF-α (clone: MP6-XT22), 1:100	Biolegend	cat: 506322
Ms CD69 PerCP-Cy5.5 (clone: H1.2F3), 1:100	BD Biosciences	cat: 551113
Ms CD62L BUV737 (clone: MEL-14), 1:100	BD Biosciences	cat: 612833
PE/Cyanine7 anti-mouse IFNγ (clone: XMG1.2), 1:100	Biolegend	cat: 505826
PE anti-human/mouse Granzyme B Recombinant (clone: QA16A02), 1:100	Biolegend	cat: 372208
PE anti-mouse CD279 (PD-1) (clone: 29F.1A12), 1:100	Biolegend	cat: 135206
PE/Cyanine7 anti-mouse CD366 (Tim-3) (clone: RMT3-23), 1:100	Biolegend	cat: 119716
Brilliant Violet 421™ anti-mouse CD3 (clone: 17A2), 1:100	Biolegend	cat: 100228
FITC anti-mouse CD3ε (clone: 500A2), 1:100	Biolegend	cat: 100305
Brilliant Violet 421™ anti-mouse CD223 (LAG-3) (clone: C9B7W), 1:100	Biolegend	cat: 125221
PerCP/Cyanine5.5 anti-mouse CD223 (LAG-3) Antibody (clone: C9B7W), 1:100	Biolegend	cat: 125211

Ms CD3e BV510 (clone: 145-2C11), 1:100	BD Biosciences	cat: 563024
Brilliant Violet 711™ anti-mouse CD8a (clone: 53-6.7), 1:100	Biolegend	cat: 100748
BUV563 anti-mouse CD4 (clone: GK1.5), 1:100	BD Biosciences	cat: 565709
BV510 anti-mouse CD4 (clone: RM4-5), 1:100	Biolegend	cat: 100553
BD Horizon™ BV786 Rat Anti-Mouse CD4 (clone: GK1.5), 1:100	BD	cat: 563331
FITC anti-mouse NK-1.1 (clone: PK136), 1:100	Biolegend	cat: 108706
Brilliant Violet 605™ anti-mouse NK-1.1 Antibody (clone: PK136), 1:100	Biolegend	cat: 108753
APC/Cy7 anti-mouse CD45.2 (clone: 104), 1:100	Biolegend	cat: 109824
Brilliant Violet 510™ anti-mouse/human CD11b (clone: 6D5), 1:100	Biolegend	cat: 101263
PE/Dazzle™ 594 anti-mouse Ly-6G/Ly-6C (clone: RB6-8C5), 1:100	Biolegend	cat: 108452
PerCP/Cyanine5.5 anti-mouse CD11c (clone: HL3), 1:100	Biolegend	cat: 117328
Alexa Fluor® 700 anti-mouse I-A/I-E (MHCII) (clone: M5/114.15.2), 1:100	Biolegend	cat: 107622
F4/80 Monoclonal Antibody (BM8), FITC, eBioscience™ (clone: BM8), 1:100	ebioscience	cat: 11-4801-81
PE/Cyanine5 anti-mouse CD19 Antibody (clone: 6D5), 1:100	Biolegend	cat: 102038
PE anti-mouse CD19 (clone: 1D3), 1:100	Biolegend	cat: 152408
Brilliant Violet 650™ anti-mouse CD25 Antibody (clone: PC61), 1:100	Biolegend	cat: 102038
APC anti-mouse CD127 (IL-7Rα) Antibody (clone: A7R34), 1:100	Biolegend	cat: 115510
Brilliant Violet 510™ anti-human CD45 (clone: HI30), 1:100	Biolegend	cat: 304035
FITC anti-human CD3 (clone: HIT3a), 1:100	Biolegend	cat: 300305
Alexa Fluor® 700 anti-human CD8a (clone: HIT8a), 1:100	Biolegend	cat: 300919
Brilliant Violet 421™ anti-human CD279 (PD-1) (clone: EH12.2H7), 1:100	Biolegend	cat: 329919
PE anti-human CD223 (LAG-3) (clone: 7H2C65), 1:100	Biolegend	cat: 369205
APC anti-human CD366 (Tim-3) (clone: F38-2E2), 1:100	Biolegend	cat: 345011
PE anti-human IFN-γ (clone: 4S.B3), 1:100	Biolegend	cat: 502508
APC anti-human TNF-α (clone: MAb11), 1:100	Biolegend	cat: 502913
Brilliant Violet 421™ anti-human/mouse Granzyme B Recombinant (clone: QA18A28), 1:100	Biolegend	cat: 396413
TruStain FcX™ PLUS (anti-mouse CD16/32) Antibody (clone: 2.4G2)	BD Biosciences	cat: 553142
*InVivo*MAb rat IgG2b isotype (clone: LTF-2)	BioXCell	cat: BE0090
*InVivo*MAb anti-mouse CD8α (clone: 2.43)	BioXCell	cat: BE0061
*InVivo*Mab anti-mouse PD-L1 (B7-H1) (clone:10F.9G2)	BioXCell	cat: BE0101

*InVivo*MAb anti-mouse PD-1 (CD279) (clone: 29F.1A12)	BioXCell	cat: BE0273
Dynabeads™ Human T-Activator CD3/CD28	Miltenyi Biotech	cat: 130-091-441
Purified anti-mouse CD3ε (clone: 145-2C11)	Biolegend	cat: 100302
Purified anti-mouse CD28 (clone: E18)	Biolegend	cat: 122002
Recombinant Human IFN-gamma Protein	R&D	cat: 285-IF-100/CF
Recombinant Murine IFN-γ	PeproTech	cat: 315-05
IFN gamma Protein, Mouse, Recombinant	Sinobiological	cat: 50709-MNAH
Cytochalasin B	Meilunbio	cat: 14930-96-2
PEI	Polysciences	cat: 23966-1
Polybrene	Sigma-Aldrich	cat: H9268
Puromycin	Sigma-Aldrich	cat: P8833
TRIzol reagent	Thermo Fisher Scientific	cat: 15596-018
HiScript II 1st Strand cDNA Synthesis Kit	Vazyme	cat: R211-02
SYBR Green Master Mix	Vazyme	cat: Q711-03
Protein A/G-conjugated beads	Thermo Fisher Scientific	cat: 53133
ClonExpressTM II One Step Cloning Kit	Vazyme	cat: C112-01
CD8a (Ly-2) MicroBeads, mouse	Miltenyi Biotech	cat: 130-117-044
CD8 MicroBeads, human	Miltenyi Biotech	cat: 130-045-201
Tumor Cell Isolation Kit, mouse	Miltenyi Biotech	cat: 130-110-187
BD Cytofix/Cytoperm™ Fixation/Permeabilization Kit	BD Biosciences	cat: 554714
Paraformaldehyde-glutaraldehyde	Leagene Biotechnology	cat: DF0139
DAPI Staining Solution	Beyotime	cat: C1005
RPMI Medium 1640 basic (1×)	Gibco	cat: C11875500BT
DMEM	Gibco	cat: 12800-017
Penicillin-Streptomycin Solution	Biological Industries	cat: 03-031-1
Fetal Bovine Serum	Gibco	cat: 10099141
Ringer's solution	LEAGENE	cat: CZ0045
Percoll	Cytiva	cat: 17089109
HEPES (1 M)	Gibco	cat: 15630-080
2-Mercaptoethanol	Gibco	cat: 21985-023
Murine IL-2	Proteintech	cat: 212-12
Human IL-2	PeproTech	cat: AF-200-02-50
Annexin V-FITC/PI	Bestbio	cat: BB-4101-50T
