# Supplementary material for: HKDC1 promotes tumor immune evasion in hepatocellular carcinoma by coupling cytoskeleton to STAT1 activation and PD-L1 expression
Source: Nat Commun. 2024 Feb 13;15:1314. doi: 10.1038/s41467-024-45712-2 (PMC10864387; doi:10.1038/s41467-024-45712-2)
Supplement: Supplementary file 3 — Reporting Summary [file 41467_2024_45712_MOESM3_ESM.pdf]

nature portfolio

Corresponding author(s): Ping Gao

Last updated by author(s): Jan 25, 2024

Reporting Summary

Nature Portfolio wishes to improve the reproducibility of the work that we publish. This form provides structure for consistency and transparency in reporting. For further information on Nature Portfolio policies, see our Editorial Policies and the Editorial Policy Checklist.

Statistics

For all statistical analyses, confirm that the following items are present in the figure legend, table legend, main text, or Methods section.

n/a	Confirmed	
☐	☒	The exact sample size (n) for each experimental group/condition, given as a discrete number and unit of measurement
☐	☒	A statement on whether measurements were taken from distinct samples or whether the same sample was measured repeatedly
☐	☒	The statistical test(s) used AND whether they are one- or two-sided<br>*Only common tests should be described solely by name; describe more complex techniques in the Methods section.*
☒	☐	A description of all covariates tested
☐	☒	A description of any assumptions or corrections, such as tests of normality and adjustment for multiple comparisons
☐	☒	A full description of the statistical parameters including central tendency (e.g. means) or other basic estimates (e.g. regression coefficient) AND variation (e.g. standard deviation) or associated estimates of uncertainty (e.g. confidence intervals)
☐	☒	For null hypothesis testing, the test statistic (e.g. F, t, r) with confidence intervals, effect sizes, degrees of freedom and P value noted<br>*Give P values as exact values whenever suitable.*
☒	☐	For Bayesian analysis, information on the choice of priors and Markov chain Monte Carlo settings
☒	☐	For hierarchical and complex designs, identification of the appropriate level for tests and full reporting of outcomes
☐	☒	Estimates of effect sizes (e.g. Cohen's d, Pearson's r), indicating how they were calculated

Our web collection on statistics for biologists contains articles on many of the points above.

Software and code

Policy information about availability of computer code

Data collection	Q-Exactive-plus mass spectrometer (Thermo Fisher Scientific) was used to collect all proteomics data.<br>Images of IHC and HE were acquired with Leica AperioCS2.<br>Images of IF staining were captured using a Zeiss 710 laser scanning confocal microscope (Zeiss).<br>Flow cytometry data were collected on a BD Fortessa or Cytek NL-3000.
Data analysis	Proteome Discovery (v 2.2) with Seaquest HT (v1.17) search engine and mascot version with Andromeda search engine (v 2.2.2) were used to analyze proteomics data.<br>Halo software (v 3.3.14) was used to analyze Images quantification of IHC and HE.<br>ImageJ software (v 1.53k) was used to analyze Images quantification of IF.<br>Flow Jo (v 10.8.1) software was used to analyze Flow cytometry data.<br>Graph Pad Prism (v 9.0).

For manuscripts utilizing custom algorithms or software that are central to the research but not yet described in published literature, software must be made available to editors and reviewers. We strongly encourage code deposition in a community repository (e.g. GitHub). See the Nature Portfolio guidelines for submitting code & software for further information.

Data

Policy information about availability of data

All manuscripts must include a data availability statement. This statement should provide the following information, where applicable:

- Accession codes, unique identifiers, or web links for publicly available datasets
- A description of any restrictions on data availability
- For clinical datasets or third party data, please ensure that the statement adheres to our policy

The transcriptomic data used in this study are available in the HCC cases in The Cancer Genome Atlas (TCGA) database under accession code phs000178 (https://www.ncbi.nlm.nih.gov/projects/gap/cgi-bin/study.cgi?study_id=phs000178.v11.p8). The GO30140 and IMbrave150 cohorts used in this study are available in the European Genome-phenome Archive (EGA) under accession code EGAD00001008130 (https://web2.ega-archive.org/dacs/EGAC00001002314). The mass spectrometry proteomics data generated in this study have been deposited to the ProteomeXchange Consortium database under accession code PXD047388 (http://proteomecentral.proteomexchange.org). The remaining data are available within the Article, Supplementary Information or Source Data file.

Research involving human participants, their data, or biological material

Policy information about studies with human participants or human data. See also policy information about sex, gender (identity/presentation), and sexual orientation and race, ethnicity and racism.

Reporting on sex and gender	Sex-independent.
Reporting on race, ethnicity, or other socially relevant groupings	N/A
Population characteristics	The normal-tumor-paired protein samples from 28 HCC patients were obtained from the First Affiliated Hospital of University of Science and Technology of China. Of the 28 liver cancer patients, 3 were women and 25 were men, with 16 patients over the age of 50.<br>The tumor RNA samples from 50 liver cancer patients were obtained from the First Affiliated Hospital of University of Science and Technology of China. Of the 28 liver cancer patients, 5 were women and 45 were men, with 25 patients over the age of 50.<br>The IHC samples from 50 liver cancer patients were obtained from Servicebio company (40 samples) and the First Affiliated Hospital of University of Science and Technology of China (10 samples). Of the 50 liver cancer patients, 9 were women and 41 were men, with 20 patients over the age of 50.
Recruitment	Liver Samples were obtained from patients diagnosed with HCC and underwent surgery at the First Affliated Hospital of the University of Science and Technology of China mainly from 2018 to 2019. Blood samples of 38 healthy individuals were obtained from the First Affliated Hospital of the University of Science and Technology of China mainly from 2018 to 2019. PBMCs seperated from blood were stored in liquid nitrogen and used after cell resuscitation.<br>For protein samples, we selected samples that dinot undergo protein degradation. For RNA samples, we used all the samples we had in our lab. For IHC samples, we uses slices with no quality problems. For PBMCs, We select cells that are in good condition after resuscitation. These operations can ensure the accuracy of the experimental results.<br>For use of these clinical materials for research purpose, prior patients' written informed consents and approval from the Institutional Research Ethics committee of the First Affiliated Hospital of university of science and Technology of china were obtained. No other biases existed in this study.
Ethics oversight	Ethical approval for the studies was obtained from the Institutional Research Ethics Committee of the First Affiliated Hospital of University of Science and Technology of China.

Note that full information on the approval of the study protocol must also be provided in the manuscript.

Field-specific reporting

Please select the one below that is the best fit for your research. If you are not sure, read the appropriate sections before making your selection.

[x] Life sciences [ ] Behavioural & social sciences [ ] Ecological, evolutionary & environmental sciences

For a reference copy of the document with all sections, see nature.com/documents/nr-reporting-summary-flat.pdf

Life sciences study design

All studies must disclose on these points even when the disclosure is negative.

Sample size	We determined the sample sizes based on preliminary studies in our laboratories or in similarly published research (Yan R. et.al., Nat Immunol. (2024); Gu XM. et. al., Nature Communications, (2023) 14:8154). For in vitro experiments, the sample sizes were enough to be detected, and we observed statistical significant difference in at least three biologically independent experiments. For animal experiments, we used six- to eight-week-old male mice (n≥5 per group) unless otherwise stated and followed the 3 R's of animal research.
Data exclusions	No data were excluded from the data set.

Replication	Each experiment was repeated at least three times independently with similar results. For animal studies, we used 5 -7 male mice (unless otherwise stated) for each different group, and the statistical significance was shown in figures. We confirmed successful replication for our reported data.
Randomization	For in vivo experiments, mice were randomly allocated to control group or treatment groups. For in vitro experiments, all samples were analyzed equally with no subsampling, and therefore was no requirement for randomization.
Blinding	The experimental conditions were not blinded in vitro experiments, since the comparisons were objective and quantitative. During the in vivo experiments, investigators were blinded to group assignments. For IHC experiments, the investigators were blinded for the clinical information of each sample prior to immunostaining analysis.

Reporting for specific materials, systems and methods

We require information from authors about some types of materials, experimental systems and methods used in many studies. Here, indicate whether each material, system or method listed is relevant to your study. If you are not sure if a list item applies to your research, read the appropriate section before selecting a response.

Materials & experimental systems

n/a	Involved in the study
☐	☒ Antibodies
☐	☒ Eukaryotic cell lines
☒	☐ Palaeontology and archaeology
☐	☒ Animals and other organisms
☐	☒ Clinical data
☒	☐ Dual use research of concern
☒	☐ Plants

Methods

n/a	Involved in the study
☒	☐ ChIP-seq
☐	☒ Flow cytometry
☒	☐ MRI-based neuroimaging

Antibodies

Antibodies used

Antibodies for western blot:
HKDC1 Polyclonal; Proteintech; cat: 25874-1-AP; lot: 00056298; 1:1000
STAT1 Polyclonal; Proteintech; cat: 10144-2-AP; lot: 00102826; 1:3000
PD-L1 Polyclonal; Proteintech; cat: 28076-1-AP; lot: 00104383; 1:3000
STAT1 (Phospho-Tyr701) Polyclonal; Sangon Biotech; cat: D155017; 1:500
STAT1 (Phospho-Ser727) Monoclonal; Abcam; cat: ab109461; lot: 10; clone number: EPR3146; 1:500
ACTA2 Monoclonal; Abcam; cat: ab124964; lot: GR303485-17; clone number: EPR5368; 1:15000
β-Actin Monoclonal; Proteintech; cat: 66009-1-Ig; lot: 10004156; 1:5000
Calnexin Polyclonal; Proteintech; cat: 10427-2-AP; lot: 00094165; 1:5000
LaminB Polyclonal; Proteintech; cat: 12987-1-AP; lot: 00061832; 1:5000
α-Tubulin Monoclonal; Proteintech; cat: 66031-1-Ig; lot: 1004185; 1:3000
HRP-conjugated anti-rabbit; Bio-Rad; cat: 170-6515; lot: L005679; 1:10000
HRP-conjugated anti-mouse; Bio-Rad; cat: 170-6516; lot: L005680; 1:10000
HA-Tag Monoclonal; Proteintech; cat: 66006-2-Ig; lot: 10011878; 1:2000
DYKDDDDK-tag Monoclonal; Proteintech; cat: 66008-3-Ig; lot: 0021526; 1:2000
GFP-tag Polyclonal; Proteintech; cat: 50430-2-AP; lot: 00105240; 1:3000
GST-tag Monoclonal; Proteintech; cat: 66001-2-Ig; lot: 10005463; 1:3000
His-Tag Monoclonal; Proteintech; cat: 66005-1-Ig; lot: L0004365; 1:5000

Antibodies for immunoprecipitation:
STAT1 Monoclonal; Abcam; cat: AB234400; lot: 1024392-12; 1ul antibody: 2mg protein
HKDC1 Polyclonal; Proteintech; cat: 25874-1-AP; lot: 00056298; 1ul antibody: 1mg protein
Flag-M2 Monoclonal; Sigma-Aldrich; cat:cat: F1804; lot: SLBK1346V; clone: M2; 1ul antibody: 1mg protein

Antibodies for immunohistochemistry:
HKDC1 Polyclonal; Proteintech; cat: 25874-1-AP; lot: 00056298; 1:1000
PD-L1 Polyclonal; Proteintech; cat: 28076-1-AP; lot: 00104383; 1:2000
STAT1 (Phospho-Ser727) Monoclonal; Abcam; cat: ab109461; lot: 10; 1:100

Antibodies for Immunofluorescence:
STAT1 Polyclonal; Proteintech; cat: 10144-2-AP; lot: 00102826; 1:50
ACTA2 Monoclonal; Abcam; cat: ab124964; lot: GR303485-17; clone number: EPR5368; 1:500
Flag-M2 Monoclonal; Sigma-Aldrich; cat: F1804; lot: SLBK1346V; clone name: M2; 1:3000

Antibodies for flow cytometry:

Ms TNF BB700; BD Biosciences; cat: 566510; lot: 9289992; clone number: MP6-XT22; 1:100
PerCP/Cyanine5.5 anti-mouse TNF-α; Biolegend; cat: 506322; lot: 13365446; clone number: MP6-XT22; 1:100
Ms CD69 PerCP-Cy5.5; BD Biosciences; cat: 551113; lot: 1004613; clone number: H1.2F3; 1:100
Ms CD62L BUV737; BD Biosciences; cat: 612833; lot: 0086470; clone number: MEL-14; 1:100
PE/Cyanine7 anti-mouse IFNγ; Biolegend; cat: 505826; lot: B311730; clone number: XMG1.2; 1:100
PE anti-human/mouse Granzyme B Recombinant; Biolegend; cat: 372208; lot: B316858; clone number: QA16A02; 1:100
PE anti-mouse CD279 (PD-1); Biolegend; cat: 135206; lot: B330031; clone number: 29F.1A12; 1:100
PE/Cyanine7 anti-mouse CD366 (Tim-3); Biolegend; cat: 119716; lot: B268015; clone number: RMT3-23; 1:100
Brilliant Violet 421™ anti-mouse CD3; Biolegend; cat: 100228; lot: B295089; clone number: 17A2; 1:100
FITC anti-mouse CD3ε; Biolegend; cat: 100305; lot: B324851; clone number: 500A2; 1:100; 1:100
Brilliant Violet 421™ anti-mouse (CD223); Biolegend; cat: 125221; lot: B297637; clone number: C9B7W; 1:100
PerCP/Cyanine5.5 anti-mouse CD223 (LAG-3) Antibody; Biolegend; cat: 125211; lot: B388626; clone number: C9B7W
Ms CD3e BV510; BD Biosciences; cat: 563024; lot: 1025799; clone number: 145-2C11; 1:100
Brilliant Violet 711™ anti-mouse CD8a; Biolegend; cat: 100748; lot: B310120; clone number: 53-6.7; 1:100
BUV563 anti-mouse CD4; BD Biosciences; cat: 565709; lot: 1088523; clone number: GK1.5; 1:100
BV510 anti-mouse CD4; Biolegend; cat: 100553; lot: B370264; clone number: RM4-5; 1:100
BD Horizon™ BV786 Rat Anti-Mouse CD4; BD; cat: 563331; lot: 3061703; clone number: GK1.5; 1:100
FITC anti-mouse NK-1.1; Biolegend; cat: 108706; lot: B311814; clonenumber: PK136; 1:100
Brilliant Violet 605™ anti-mouse NK-1.1 Antibody; Biolegend; cat: 108753; lot: B376036; clone number: PK136; 1:100
APC/Cy7 anti-mouse CD45.2; Biolegend; cat: 109824; lot: B338088; clone number: 104; 1:100
Brilliant Violet 510™ anti-mouse/human CD11b; Biolegend; cat: 101263; lot: B288417; clone number: 6D5; 1:100
PE/Dazzle™ 594 anti-mouse Ly-6G/Ly-6C; Biolegend; cat: 108452; lot: 1124024; clone number: RB6-8C5 ; 1:100
PerCP/Cyanine5.5 anti-mouse CD11c; Biolegend; cat: 117328; lot: 9304609; clone number: HL3; 1:100
Alexa Fluor® 700 anti-mouse I-A/I-E; Biolegend; cat: 107622; lot: B264454; clone number: M5/114.15.2; 1:100
F4/80 Monoclonal Antibody (BM8), FITC, eBioscience™; ebioscience; cat: 11-4801-81; lot: 3061703; clone: BM8; 1:100
PE/Cyanine5 anti-mouse CD19 Antibody; Biolegend; cat: 102038; lot: B379247; clone: 6D5; 1:100
PE anti-mouse CD19; Biolegend; cat: 152408; lot: B371992; clone: 1D3; 1:100
Brilliant Violet 650™ anti-mouse CD25 Antibody; Biolegend; cat: 135012; lot: ; clone: PC61; 1:100
APC anti-mouse CD127 (IL-7Rα) Antibody; Biolegend; cat: 115510; lot: ; clone: A7R34; 1:100
Brilliant Violet 510™ anti-human CD45; Biolegend ; cat: 304035; lot: B360609; clone number: HI30; 1:100
FITC anti-human CD3; Biolegend; cat: 300305; lot: B319960; clone number: HIT3a; 1:100
Alexa Fluor® 700 anti-human CD8a; Biolegend; cat: 300919; lot: B256904; clone number: ; 1:100
Brilliant Violet 421™ anti-human CD279 (PD-1); Biolegend; cat: 329919; lot: B354269; clone number: EH12.2H7; 1:100
PE anti-human CD223 (LAG-3); Biolegend; cat: 369205; lot: B348425; clone number: 7H2C65; 1:100
APC anti-human CD366 (Tim-3); Biolegend; cat: 345011; lot: B335176; clone number: F38-2E2; 1:100
PE anti-human IFN-γ; Biolegend; cat: 502508; lot: B352971; clone number: 4S.B3; 1:100
APC anti-human TNF-α; Biolegend; cat: 502913; lot: B351607; clone number: MAB11; 1:100
Brilliant Violet 421™ anti-human/mouse Granzyme B Recombinant; Biolegend; cat: 396413; lot: B355193; clone number: QA18A28; 1:100

Validation

All antibodies were purchased from commercial suppliers including Proteintech, Abcam, Sangon Biotech, Sigma-Aldrich, Bio-Rad, R&D, Biolegend, BD Biosciences with validation data, statement and applicable citations available on product listings for all antibodies (see individual catalog numbers). Additionally, all antibody were titrated for optimal dilution in the assay. We also validated the antibodies by western blot using specific targeting shRNAs or over-expressing vectors.

Eukaryotic cell lines

Policy information about cell lines and Sex and Gender in Research

Cell line source(s)

Human HEK293T, HepG2, Hep3B and mouse Hepa1-6 cell lines were purchased from ATCC. Mouse Hep55.1c cell line was purchased from the BioVector NTCC Inc.

Authentication

Cell line identities were confirmed by STR profiling.

Mycoplasma contamination

All cell lines were tested routinely to make sure they are negative for mycoplasma contamination by Mycoplasma PCR detecting method

Commonly misidentified lines
(See ICLAC register)

No commonly misidentified cell lines were used.

Animals and other research organisms

Policy information about studies involving animals; ARRIVE guidelines recommended for reporting animal research, and Sex and Gender in Research

Laboratory animals

Six- to eight-week-old mice (C57BL/6JGpt; GemPharmatech company);
HKDC1−/− mice (C57BL/6JGpt) were generated using CRISPR genome editing (target sequence: 5′-CACAGACGTGGTGAACCGCC-3′) were obtained from the animal facility of the University of Science and Technology of China.

	All animals were housed at a suitable temperature (22–24 °C) and humidity (40–70%) under a 12/12-h light/dark cycle with unrestricted access to food and water for the duration of the experiment.
Wild animals	No wild animals were used in this study.
Reporting on sex	Sex-independent.
Field-collected samples	No field-collected samples were used in this study.
Ethics oversight	All animal studies were conducted with approval from the Animal Research Ethics Committee of South China University of Technology.

Note that full information on the approval of the study protocol must also be provided in the manuscript.

Clinical data

Policy information about clinical studies
All manuscripts should comply with the ICMJEguidelines for publication of clinical research and a completedCONSORT checklist must be included with all submissions.

Clinical trial registration	N/A
Study protocol	N/A
Data collection	N/A
Outcomes	N/A

Flow Cytometry

Plots

Confirm that:

- [x] The axis labels state the marker and fluorochrome used (e.g. CD4-FITC).
- [x] The axis scales are clearly visible. Include numbers along axes only for bottom left plot of group (a 'group' is an analysis of identical markers).
- [x] All plots are contour plots with outliers or pseudocolor plots.
- [x] A numerical value for number of cells or percentage (with statistics) is provided.

Methodology

Sample preparation	Single cell suspensions were prepared from cells in culture or tumors of HCC-bearing mice. For tumor samples from HCC-bearing mice, single cell suspension was obtained by rapid and gentle stripping, physical grinding and filter filtration. Single lymphocytes were blocked with CD16/CD32 antibody (BioLegend, CA, USA, 553142) and stained with indicated fluorochrome-conjugated antibodies for 30 min at 4°C. To incubate the following antibodies: anti-IFNγ, anti-TNFα and anti-Granzyme B, cells should be permeabilized with a Cytofix/Cytoperm Kit (BD Biosciences, 554714). The antibodies used in this study are listed in supplementary information, Table S3.
Instrument	BD FACSAria SORP, BD FACSCelestaTM Flow Cytometer
Software	Flow Jo 10.0 software
Cell population abundance	10,000 cells were analyzed for each sample.
Gating strategy	For all experiments, cells were first gated by FSC/SSC to exclude debris, followed by gating SSC-A and SSC-H to eliminate non-singlets. Then, target cell population for further analysis were gated by cell surface marker (e.g. CD45, CD3, CD8). Isotype control antibodies were used as negative controls.

- [ ] Tick this box to confirm that a figure exemplifying the gating strategy is provided in the Supplementary Information.
